# An Improved Machine Learning-Based Approach to Assess the Microbial Diversity in Major North Indian River Ecosystems

**DOI:** 10.3390/genes14051082

**Published:** 2023-05-14

**Authors:** Nalinikanta Choudhury, Tanmaya Kumar Sahu, Atmakuri Ramakrishna Rao, Ajaya Kumar Rout, Bijay Kumar Behera

**Affiliations:** 1 ICAR—Indian Agricultural Research Institute, New Delhi 110012, India; nalini.ctc.7@gmail.com; 2 ICAR—Indian Agricultural Statistics Research Institute, New Delhi 110012, India; 3 ICAR—Indian Grassland and Fodder Research Institute, Jhansi 284003, India; tanmayabioinfo@gmail.com; 4 Indian Council of Agricultural Research (ICAR), New Delhi 110001, India; 5 ICAR—Central Inland Fisheries Research Institute, West Bengal 700120, Indiabeherabk18@yahoo.co.in (B.K.B.); 6 Rani Lakshmi Bai Central Agricultural University, Jhansi 284003, India

**Keywords:** metagenomics, K-Means clustering, support vector machine, binning, river sediment

## Abstract

The rapidly evolving high-throughput sequencing (HTS) technologies generate voluminous genomic and metagenomic sequences, which can help classify the microbial communities with high accuracy in many ecosystems. Conventionally, the rule-based binning techniques are used to classify the contigs or scaffolds based on either sequence composition or sequence similarity. However, the accurate classification of the microbial communities remains a major challenge due to massive data volumes at hand as well as a requirement of efficient binning methods and classification algorithms. Therefore, we attempted here to implement iterative K-Means clustering for the initial binning of metagenomics sequences and applied various machine learning algorithms (MLAs) to classify the newly identified unknown microbes. The cluster annotation was achieved through the BLAST program of NCBI, which resulted in the grouping of assembled scaffolds into five classes, i.e., bacteria, archaea, eukaryota, viruses and others. The annotated cluster sequences were used to train machine learning algorithms (MLAs) to develop prediction models to classify unknown metagenomic sequences. In this study, we used metagenomic datasets of samples collected from the Ganga (Kanpur and Farakka) and the Yamuna (Delhi) rivers in India for clustering and training the MLA models. Further, the performance of MLAs was evaluated by 10-fold cross validation. The results revealed that the developed model based on the Random Forest had a superior performance compared to the other considered learning algorithms. The proposed method can be used for annotating the metagenomic scaffolds/contigs being complementary to existing methods of metagenomic data analysis. An offline predictor source code with the best prediction model is available at (https://github.com/Nalinikanta7/metagenomics).

## 1. Introduction

Rivers are an essential source of development not only for mankind, but also for the aquatic ecosystems consisting of microbes to fish. Microbial communities have a crucial role in aquatic ecology as they regulate biogeochemical cycles, decompose organic matter and provide a food source for higher trophic levels. Microbes are omnipresent and abundantly found in river water and sediments. Thus, there is a need to explore the diversity and density of microbes to analyze their impact on human as well as fish health [1]. The Ganga and the Yamuna rivers, the most important river ecosystems in northern India and the third-largest aquatic ecosystem globally, discharge water into the sea [2]. These two rivers, along with their tributaries, are the heart of water resources in northern India and they hold a wide diversity of microbes. With the rapid advancements in sequencing techniques, metagenomics has been extensively applied to study the microbial communities of aquatic, soil and other ecosystems.

In metagenomics, the genomes of several microbes are analyzed from the sediment samples collected from a particular niche that is otherwise difficult to culture in a laboratory [3]. It includes research avenues such as systems biology, bioinformatics [4] and statistics. One of the crucial steps in metagenomic studies is the process of binning that allows the assignment of microbial assembled contigs to their respective phylogenetic groups [5]. The available binning techniques are categorized into two major groups, i.e., composition-based [6] and similarity-based methods [7]. Among the sequence similarity-based binning methods, MEGAN [8], Phylopythia [9], BLAST [10,11], etc., are popular, and they use a sequence alignment that depends on a known genome database. Therefore, similarity-based binning algorithms are time-consuming. On the other hand, composition-based binning takes less execution time by considering sequence compositional features such as GC content, nucleotide frequencies, k-mer (di, tri, tetra nucleotides) frequencies, composition–transition–distribution (CTD) features, etc. These features encode the sequences and categorize them into different taxonomic groups. Methods such as MetaCAA [12] and self-organizing maps [13] were previously used to cluster the metagenome sequence data based on the sequence as mentioned above, compositional features. All these methods are rule-based and require higher computational resources such as memory and execution time, whereas MegaR [14] and MetAML [15] are recently developed machine learning-based approaches which can classify the metagenome data efficiently.

In-depth taxonomic profiles and roles of bacterial, viral and planktonic communities, as well as population interactions, are now best understood through metagenomics [16,17]. The density and diversity of the microbial population from the sediments of the Ganga and the Yamuna rivers are reported by Behera et al. [18], Sahu et al. [17] and Samson et al. [19]. However, a large number of microbes from these river ecosystems remained unclassified. Behera et al. [18] used Kaiju [20] for binning purposes, a rule-based method (which is neither a supervised nor an unsupervised machine learning technique), and classified a limited number of microbes. In the metagenomic study, supervised MLAs perform well but consume more time as compared to unsupervised MLAs due to the diversity of organisms present [21]. The supervised learning requires labeled data and focuses on predicting a target variable, while unsupervised learning works on unlabeled data to uncover patterns and relationships within the data. The accuracy of MLAs can be improved by balancing the number of microbial sequences present in each class. Therefore, a mixture of unsupervised and supervised learning algorithms is required to develop a faster method with improved accuracy for microbial diversity analysis.

Here, we propose a method involving iterative K-Means clustering for binning metagenomic data and MLAs for developing a classifier to predict the unknown microbes. The iterative K-Means clustering is an unsupervised learning algorithm that operates on unlabeled datasets, with the aim of dividing the data points into distinct clusters. The number of clusters to be created, which is denoted by K, is specified prior to the execution of the algorithm. This iterative algorithm repeatedly partitions the data points into K clusters based on their similarity, until the clusters are well defined and the partitioning converges. In this study, the iterative K-Means clustering assigns the unannotated assembled scaffolds into the clusters of known scaffolds based on their distances from the prebuilt cluster centroid of the annotated scaffold data. The MLAs were applied on the labeled data generated from the K-Means clustering method, and subsequently, the performance matrices were derived. The performances of the MLAs were improved by using the best set of parameters obtained through the grid search technique. The proposed model can assist in the improvement of the classification of microbial populations present in aquatic ecosystems into different groups, such as bacteria, archaea, eukaryota, viruses and others, etc., based on metagenomic data. A portable program is also scripted here to help metagenomic scientists and researchers classify the microbial diversity in any ecosystem.

## 2. Materials and Methods

### 2.1. Data Acquisition

The sediment samples from the Ganga River at Kanpur and Farakka locations and the Yamuna River at Delhi were collected from three sites each. Using a genomic DNA (gDNA) isolation kit, gDNA was extracted from 250 mg of river sediment samples (Nucleospin Soil, Takara, Mountain View, CA, USA). Subsequently, the high-quality isolated DNA was subjected to 1% agarose gel (loaded 5 μL) electrophoresis at 110 V for 30 min and acceptable quality of sediment gDNA was used later on for next-generation library preparation. The quality of the gDNA was determined based on A260/280 ratio, where 1 μL from each sample was loaded in Nanodrop 2000. The concentration of gDNA was further assessed using Qubit 3.0 Fluorometer developed by Thermo Fisher Scientific, Waltham, MA, USA The DNA extracted from the collected samples was sequenced by Illumina Trueseq technology [22]. The data was generated at ICAR-Central Inland Fisheries Research Institute, West Bengal, India. The sequencing depths for the metagenomic samples from Kanpur, Farakka and Delhi were ~32 million reads/sample, ~37 million reads/sample and ~66 million reads/sample, respectively.

### 2.2. Preprocessing and Genome Assembly

**Figure 1:**
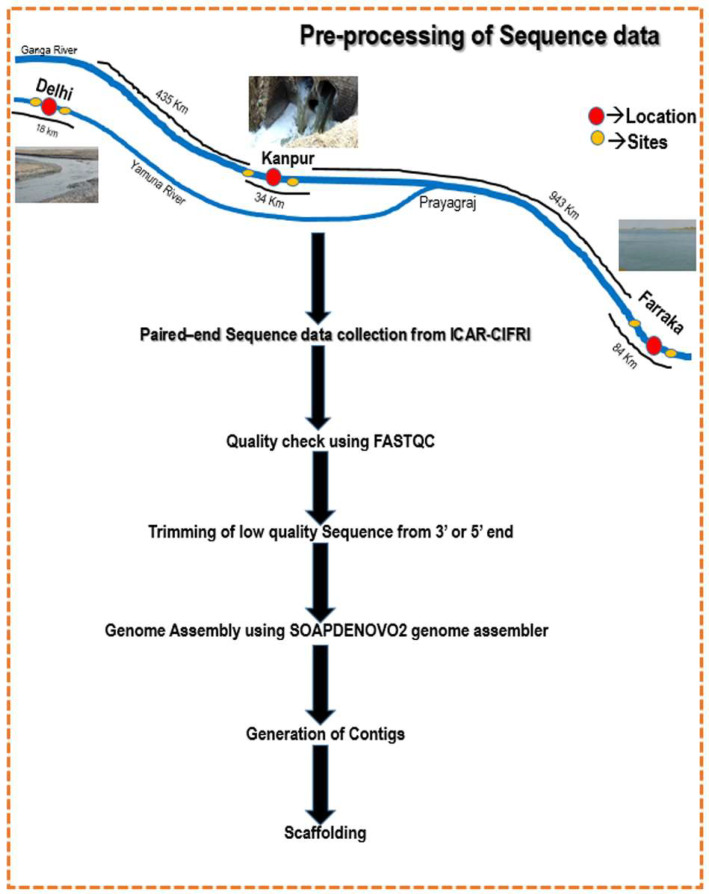
Preprocessing steps for metagenomic samples collected from Delhi, Kanpur and Farakka locations.

The Illumina Trueseq (Illumina, San Diego, CA, USA) paired-end raw sequence reads were initially checked for quality by using FASTQC (version 0.11.9) [23]. The low-quality reads (Phred score < 33) and vector sequences were trimmed out using the Trimmomatic tool (version 0.40) [24]. The trimmed sequences were then assembled using SOAP-DENOVO2 [25] that is capable of assembling Illumina short reads from metagenomic samples. The steps for preprocessing the sequence data are illustrated in Figure 1. The extracted scaffolds were aligned to the ‘nr’ database [26] of NCBI by using offline BLAST [27]. The scaffold hits with the nr-database were designated as Scaffolds with Hits (SWH), whereas the scaffolds left without hits were named as Scaffolds without Hits (SWoH). The genus and super-kingdom of SWH using ENTRENZ gateways were identified by using an in-house R-script (provided in the GitHub account). The identical sequences and sequences with non-standard residues were filtered out from the dataset using the in-house scripts, and remaining sequences were used for further analysis.

### 2.3. Feature Extraction

The feature extraction from the SWH and SWoH scaffold sequences was performed using BioSeqClass [28,29], Seqinr [30] and BioStrings [31] packages of Bioconductor using a high-end server with 16 nodes of Linux-based cluster available at ICAR-Indian Agricultural Statistics Research Institute, New Delhi, India that is configured with 192 cores of processor. Four features including *CTD* (composition, transition and distribution), fragment composition, gap-pair composition, and CKSAAP (composition of k-spaced amino acid pairs) were combined and used in this study to generate a numeric vector of length 258. All of these features are briefly described in the following subsections.

#### 2.3.1. CTD (Composition, Transition and Distribution) Feature

The sequences are encoded based on their composition (C), transition (T) and distribution (D) properties by using the CTD [32] features. C is the number of nucleotides of a particular property divided by the total number of nucleotides. T characterizes the percent frequency with which nucleotides of a different property follow the nucleotide of a particular property. D measures the chain length within which the first 25, 50, 75 and 100 nucleotides of a specific property are located. This sequence encoding feature returns a matrix with 30 columns.

#### 2.3.2. Fragment Composition Feature

In the FragmentComposition [33] features, the sequences are encoded based on the frequency of k-mer sequence fragments. It returns a matrix with 84 columns.

#### 2.3.3. Gap-Pair Composition Feature

The sequences are encoded based on the frequency of g-spaced nucleotide pairs based on *GapPairComposition* [34] features. It returns a matrix with 48 columns.

#### 2.3.4. CKSAAP (Composition of K-Spaced Amino Acid Pairs) Features

In *featureCKSAAP* [35], sequences are encoded based on the frequency of k-spaced amino acids/base pairs. It returns a matrix with 96 columns.

### 2.4. Clustering for Binning

#### 2.4.1. K-Means Clustering

**Figure 2:**
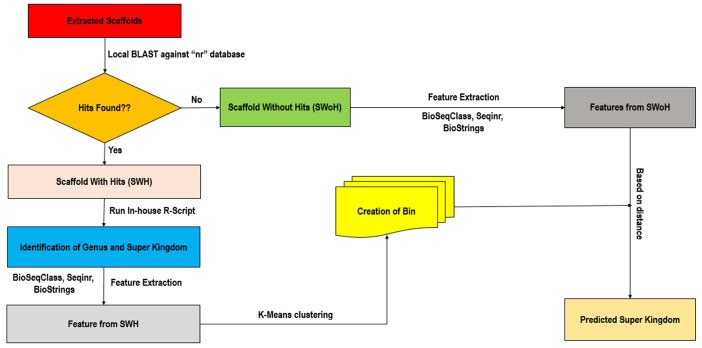
Flow chart showing the steps of iterative K-Means algorithm followed to cluster the contigs generated from the collected metagenomic samples.

The execution of the K-Means clustering method was performed in R [36]. It attempts to create the intra-cluster datasets as related as possible and create the clusters as distinct as possible. The assignment of new data points to a prebuilt cluster such that the sum of squared distance between the data points and the prebuilt cluster’s centroid is minimal. The variation within the clusters should be minimum and the divergence between the clusters should be maximum. The algorithm initially examines as a group of “k” centroids, and each centroid connects to each scaffold in the dataset to the adjacent centroid based on the Euclidean distance [37] measure, which is given as follows:

$$\mathrm{Euclidean}\;\mathrm{distance}\;(X,\;Y)=\sqrt{\sum_{i=1}^{n}{\left(x_{i}-y_{i}\right)}^{2}}$$

where (X, Y) are the coordinates of a point or instance with n number of features and *x_i_*, *y_i_* denote the *i*th element of the corresponding (X, Y) coordinate. The K-Means clustering implemented here is depicted in Figure 2.

#### 2.4.2. Evaluation of Clusters

The performance of the clusters was evaluated through a silhouette plot that describes how well the samples are clustered with others. The average distance between an observation and all other observations in the same cluster is measured as mean intra-cluster distance and denoted by *a*. The average distance between an observation with all other observations of the nearest neighbor cluster is calculated as mean inter-cluster distance and denoted by *b*. Then, the silhouette score [38] is defined as follows:

$$S_{i}=\frac{b\left(i\right)-a(i)}{max\{a\left(i\right),b\left(i\right)\}}$$

where *S*(*i*) = silhouette coefficient of the data point “*i*”. *a*(*i*) = mean distance of one data point from all other data points in the clusters to which “*i*” belongs and *b*(*i*) = least mean distance from “*i*” to all clusters to which “*i*” does not belong.

The range of silhouette coefficient ranges from −1 to 1. A score of 1 indicates that the observation “*i*” is highly dense within the cluster to which it belongs to, but at a far distance from other clusters. A score of −1 denotes a worst value be a part of a cluster and 0 denotes the overlapping of clusters.

### 2.5. Machine Learning for Classification

Here, six MLAs viz., support vector machine, Random Forest, Gradient Boosting decision tree, XGBoost, AdaBoost and BiLSTM were evaluated with a considered set of features described earlier under “Feature Extraction” sub-section. These MLAs were widely used in earlier studies to solve various biological problems, and thus, they are considered here in this study. Subsequently, performances of these MLAs were compared based on performance metrics and the best prediction model was used to develop the classifier. These MLAs are described briefly in the following subsections. Further, the advantages and disadvantages of the MLAs used in this study are given in Table S1.

#### 2.5.1. Support Vector Machine

A support vector machine (SVM) is a non-parametric and statistically robust supervised machine learning algorithm [39,40]. It works based on the principle of structural risk minimization and can handle high-dimensional noisy datasets. Its performance greatly depends on the kernel function and hyperplane type, which can discriminate the observations using the optimal linear technique. The SVM was implemented using the scikit-learn Python package [41].

#### 2.5.2. Random Forest

Random Forest is an ensemble-based classifier developed by Breiman and Cutler [42]. The method involved in RF combines two approaches, viz., Breiman’s bagging predictors [43] and random feature selection [44], to construct a set of decision trees. A class is assigned to an observation based on majority rule, i.e., the observation is assigned to a class which appears in a majority of the decision trees. The RF was implemented here using the “sklearn ensemble” library of Python. Random Forest (RF) technique reduces the variance by averaging several decision trees.

#### 2.5.3. Gradient Boosting Decision Tree

GBDT is an ensemble-boosting integrated learning model, also known as Gradient Boosting Machine (GBM), based on greedy function approximation [45]. It uses multiclass logistic likelihood for classification. It fits a decision tree on the residual errors, due to which it is called a gradient. The GBDT classifier was implemented through the “sklearn ensemble” library of Python.

#### 2.5.4. XGBoost

XGBoost is a widely used end-to-end tree-boosting MLA for classification. It is implemented with the “sklearn” Python package. It creates parallel tree enhancements to solve the machine learning problems such as regression, classification and ranking [46]. Here, XGBoost was implemented through the xgboost package available in Python.

#### 2.5.5. AdaBoost

AdaBoost (Adaptive Boosting) [47], an ensemble MLA, is generally used to increase the classifier efficiency. It is an iterative approach where the classifier learns from the weak classifier’s errors, making them stronger. It applies weights to specific samples. AdaBoost was implemented here using the “sklearn ensemble” library of Python.

#### 2.5.6. BiLSTM

Bidirectional Long Short-Term Memory (BiLSTM) [48] is a deep learning algorithm which consists of two independent Recurrent Neural Networks (RNNs) where the bidirectional run inputs in two ways, one unidirectional from past to future and the other unidirectional from future to past. BiLSTM was implemented here using the “Tensorflow.keras.layer” library of Python.

### 2.6. Training and Validation

**Figure 3:**
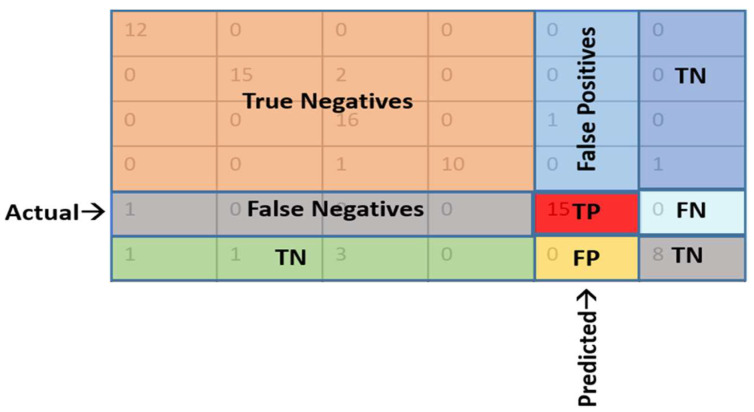
The confusion matrix for multiclass classification.TP: truly predicted into positive class, TN: truly predicted into negative class, FP: falsely predicted into positive class and FN: falsely predicted into negative class.

The extracted and combined features with a well-labelled SWH dataset were used to train the models of the machine learning approaches (MLA). Here, 80% of the data was used for training, and the remaining 20% was used for testing the machine learning classifiers. SVM was trained using the “sklearn” library [49] of Python with the Radial Basis function (RBF) as kernel function. A grid search of parameters was carried out to find the best parameter set with which the model was developed. For training the RF model, a grid search of parameters was also performed based on which the “n_estimators” parameter was set as 20, the criterion parameter was selected as “Gini” and the rest of the parameters were kept as default. The grid search of parameters was also performed for the GBDT, XGBoost, AdaBoost and BiLSTM, and the best set of parameters was selected (Table 1). The performance of all the MLA models was validated using a ten-fold cross-validation technique [50]. The performance metrics such as accuracy, precision, F1 Score, sensitivity and area under curve-receiver operating characteristics (AUC–ROC) were calculated as an average of over the ten-folds. The formulae of accuracy, precision, sensitivity and F1 Score aregiven as follows:

$$\mathrm{Accuracy}=\frac{TP+TN}{TP+TN+FP+FN}$$

$$\mathrm{Precision}=\frac{TP}{TP+FP}$$

$$\mathrm{Sensitivity}\;\mathrm{or}\;\mathrm{Recall}=\frac{TP}{TP+FN}$$

$$F1\;\mathrm{Score}=\frac{2\times Precison\times Recall}{Precision+Recall}$$

where *TP* = True Positive, *TN* = True Negative, *FN* = False Negative and *FP* = False Positive. Further, a test dataset was prepared from 20% of the datasets used to validate the performance of the models. Confusion matrices [51] were made with limited decision boundaries from validation results to calculate the performance metrics. The confusion matrix for multiclass classification is given in Figure 3. The accuracy estimation was carried out by both macro averaging and weighted averaging techniques, where macro averaging is a simple arithmetic mean of all metrics across classes, whereas in the weighted average method, each score is multiplied by the number of occurrences of each class and divided by the total number of samples.

### 2.7. Comparison with the State-of-the-art Tools

The proposed prediction model was compared with the MegaR and MetAML for the classification of microbial diversity of the metagenomic dataset. MegaR and MeAML reported RF as the best MLA in metagenomics data classification. It was also reported that the accuracy of these two RF-based machine learning classification models were 0.6683 and 0.6640, respectively. Thus, these two methods were compared with the proposed method by using their test dataset.

## 3. Results

### 3.1. The Metagenome Assembly

The assembly statistics for the contigs and scaffolds generated from the assembly of trimmed and filtered reads are given in Table 2. The metagenome data of the Delhi location was found to contain the highest number of contigs, followed by the Farakka and Kanpur locations. However, the highest number of scaffolds was observed for the metagenomic data collected from the Kanpur location, followed by Delhi and Farakka. Furthermore, the longest scaffold was also found for the Kanpur location. The average scaffold length for the Kanpur and Delhi locations was at par, whereas the average scaffold length for Farakka was between 100 to 150 bases less than that of the other two locations. The N50 parameter at Farakka and Delhi were observed to be equal, whereas the N50 for Kanpur was 18 bases lesser than that of Farakka and Delhi. The N90 parameter for all three locations was found to fall within the range of 90–100.

### 3.2. Diversity of Microbial Population

The microbes identified from metagenomic scaffolds were classified into cellular and non-cellular organisms. Some microbes were found explicitly in the Ganga and the Yamuna rivers independently. The microbial diversity of these two rivers is given in Table 3. Out of 162,836 scaffolds generated from the metagenomic data of the three locations, 99,819 scaffolds were found classified (~61.30%) into different microbial clusters. The remaining 63,017 scaffolds were left unclassified (~38.7%). Among the classified scaffolds, the bacterial scaffolds were the most dominated (83.36%) followed by the archaeal scaffolds (8.97%), the eukaryota scaffolds (7.21%) and the viral scaffolds (0.46%). It was observed that bacterial species such as *Pseudomonas* sp. and *Streptomyces* sp. were found to be abundant in the Ganga River at the Farakka and Kanpur locations, whereas *Dechloromonas* sp. and *Paraburkholderia* sp. were predominantly observed in the Yamuna River at the Delhi location. Further, it was also noticed that the proportion of *Pseudomonas* sp. and *Streptomyces* sp. are relatively lower at Farakka as compared to Kanpur. The archaeal species such as *Natrinema* sp., *Nitrososphaera* sp. and *Cenarchaeum* sp. were seen to be plenteous at the Farakka location, whereas *Haloterrigena* sp., *Aeropyrum* sp. and *Haloarcula* sp. were observed to be highly abundant at the Kanpur location. Though *Thermococcus* sp. was noticed at both the Farakka and Delhi locations, it was found to be more abundant in Delhi than the Farakka site. Other archaeal species such as *Haloferax* sp., *Halobacterium* sp. and *Halogeometricum* sp. were seen to be present in the Yamuna River at the Delhi location. The abundance of eukaryotic species such as *Paracoccus* sp., *Lobosporangium* sp.and *Parastrongyloides* sp. was noted to be higher at the Farakka location, whereas *Cyclotella* sp., *Timema* sp. and *Aspergillus* sp. were observed to be elevated at the Kanpur location. However, the eukaryota *Gaeumannomyces* sp., *Phoenix* sp. and *Strongyloides* sp. were found predominantly at the Yamuna River of Delhi. The viral species such as *Variovorax* sp. and *Roseovarius* sp. were observed to be widespread across all three sites; however, their abundance was found to be less at Farakka as compared to Kanpur and Delhi.

### 3.3. Binning of Metagenomic Data

**Figure 4:**
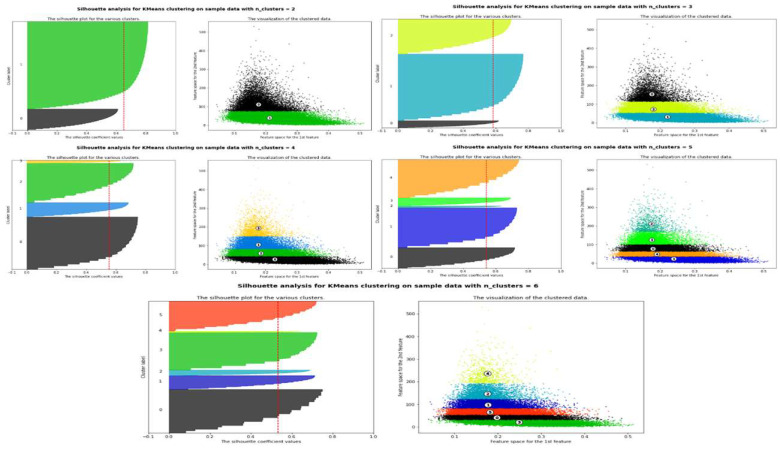
The Silhouette plot for the putative number of clusters.

Using K-Means clustering, five different clusters representing bacteria, archaea, eukaryota, viruses and others were obtained, annotated through BLAST with a cutoff similarity >99%. The clusters evaluated through silhouette plots revealed the stability of the silhouette score for five different clusters (Figure 4). The width of each silhouette plot in each cluster is a deciding factor in calculating the optimal clusters to consider. The silhouette scores for two, three, four, five and six clusters were observed to be 0.6501, 0.5826, 0.5549, 0.5424 and 0.5419, respectively. Further, the absolute differences between clusters two and three, three and four, four and five and five and six were found to be 0.0675, 0.0277, 0.0125 and 0.0005, respectively. A non-significant change is observed between the silhouette scores between five and six clusters. Therefore, five clusters were identified to be an optimal number of clusters, whereas two, three and four clusters seem to be suboptimal. The clusters were annotated based on the annotation of the individual sequences found in each cluster obtained from the BLAST results.

### 3.4. Performance of Machine Learning Algorithms at Different Locations

#### 3.4.1. Overall Prediction Performance

The annotated clusters were used as the input data for training and validating the MLA models. The performance metrics of the MLAs are given in Table 4 for different locations. It was found that the accuracy of the SVM for the Delhi location is 86%, which is higher than the other two locations. The precision and recall of the SVM for Delhi are 81% and 79%, respectively, which are also higher than that of the other two locations. However, the F1 Score at the Farakka location is found to be 80% higher than the other locations. The AUC–ROC of the SVM for Delhi was observed to be 86%, which is greater than that of Farakka and Kanpur. As far as the performance of the RF is concerned, the accuracy, precision, recall and AUC–ROC values for Delhi were noticed to be 88%, 85%, 80% and 87%, respectively, which are higher than that of Farakka and Kanpur. The F1 Score of the RF for Farakka and Delhi are found to be equivalent (i.e., 80%), whereas it was a bit lower for Kanpur. The accuracy of the GBDT for Delhi was 85%, which is 5% higher than that of the Farakka and Kanpur locations. Further, the recall of the GBDT for the Kanpur location is 82%, which is higher than the recall observed for the other two locations and the highest AUC–ROC of 81% was observed at Delhi. The XGBoost performed well for Delhi, with the highest values for precision, recall and F1 Score; however, it showed the highest accuracy for Farakka and the highest AUC–ROC for Kanpur. It is noted that the accuracy of AdaBoost at Kanpur was found to be 84%, which is higher than the accuracy observed for the other two locations. However, the precision and recall were observed to be 84% and 81%, respectively, for Delhi, and the highest F1 Score was observed for the Farakka and Delhi datasets (80%). Further, the AUC–ROC of AdaBoost was found to be the highest (78%) for the Farakka dataset. The BiLSTM performed better with the Kanpur dataset with highest values for recall, F1 Score and AUC–ROC. However, the highest accuracy (81%) at Farakka and the highest precision at Farakka and Delhi (80%) were also observed.

#### 3.4.2. Class-Wise Prediction Performance

As we have implemented a multiclass classifier to represent five different classes of microorganisms, i.e., bacteria, archaea, eukaryota, viruses and others, the MLAs were compared in terms of class-wise prediction performance (Table 5, Table 6 and Table 7) using metagenomic data from all considered locations. The SVM classifier confirmed the highest accuracy of 76% for the virus class and the highest AUC–ROC of 81% for the archaea class with the metagenomic dataset of Farakka (Table 5). In the case of Kanpur, the SVM showed the highest accuracy of 78% and AUC–ROC of 86% for the eukaryota and virus classes, respectively (Table 6). However, with the Delhi location dataset, the classifier showed the highest values for accuracy, precision, recall, F1 Score and AUC–ROC for the virus class (Table 7). The RF performed well for the virus class with the highest accuracy of 85% and the highest AUC–ROC (85%) for the bacteria and archaea classes with the Farakka dataset. RF also confirmed the highest accuracy and AUC–ROC for the virus class with both the Kanpur (80%, 87%) and Delhi (84%, 87%) datasets. The GBDT confirmed the highest values for all the considered performance metrics with the Farakka dataset and the highest accuracy (81%) and AUC–ROC value (81%) with the Delhi dataset. However, with the Kanpur dataset, it showed the highest accuracy (81%) for the virus class and the highest AUC–ROC (80%) for the archaea class.

As far as the boosting-based classifiers are concerned, the XGBoost showed the highest accuracy for the virus class (82%) and the highest AUC–ROC (75%) in predicting eukaryota with the Farakka dataset. It confirmed the highest accuracy (79%) and AUC–ROC (81%) in predicting the archaea class with the Kanpur dataset. Further, in the case of the Delhi dataset, the XGboost showed the highest accuracy (81%) for the virus class and the highest AUC–ROC (81%) for the archaea class. The AdaBoost performed well in predicting the archaea class with the Farakka dataset with an accuracy of 81% and AUC–ROC of 74%. However, it performed well for bacteria and eukaryota with the Kanpur dataset (Table 6), and for archaea, virus and eukaryota with the Delhi dataset (Table 7). The BiLSTM, a deep learning-based algorithm, exhibited the highest accuracy (82%) for eukaryota and highest AUC–ROC (75%) for the bacteria class with the Farakka dataset. However, it showed the highest values for all performance metrics in predicting the instances of the archaea class with the Kanpur dataset (Table 6) and the virus class with the Delhi dataset (Table 7).

### 3.5. Comparison of Different Classification Models

**Figure 5:**
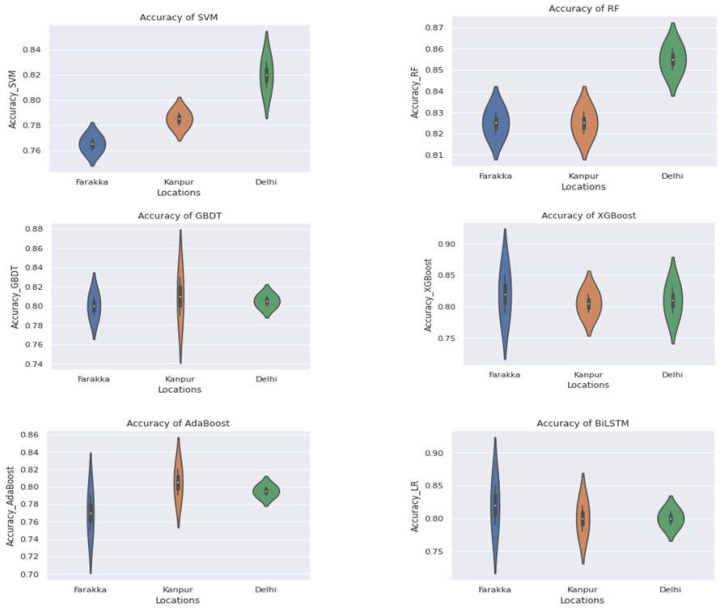
Violin plot showing accuracy of SVM, RF, GBDT, XGBoost, AdaBoost and BiLSTM at three different locations.

**Figure 6:**
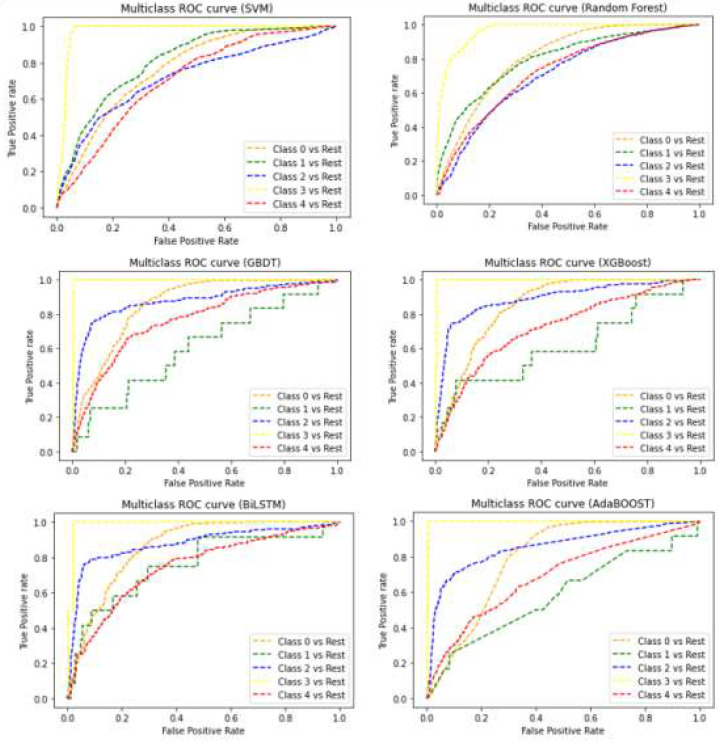
ROC curves for SVM, RF, GBDT, XGBoost, AdaBoost and BiLSTM of five different classes of microbes such as bacteria, archaea, eukaryota, fungi, viruses and other microbes represented as classes 0–4, respectively.

The performance comparison of MLAs at three different locations as a violin plot is given in Figure 5. The width and height represent the frequency of the density plot and the coverage of the maximum and minimum values of the accuracy, respectively. It is noticed that the overall accuracy, precision and AUC–ROC of the RF with Delhi dataset are highest compared to the other MLAs, irrespective of location. The ROC (Receiver Operating Characteristic) curves for the best models of the SVM, RF, GBDT, XGBoost, AdaBoost and BiLSTM are shown in Figure 6. The RF confirmed the highest AUC–ROC (87%) and highest accuracy (88%) for the Delhi location, which is also highest irrespective of location and methods. With the recall, as well as the F1 Score of 80%, the best RF model was observed for Delhi. All models were found to perform well with the Delhi dataset. The performance of the SVM follows the RF with the Delhi dataset. The RF was also found to perform suitably with the Kanpur and Farakka datasets when all performance metrics were taken into consideration. The performance metrics of the MLAs with the datasets from the considered locations implies that the RF is more suitable than the SVM, GBDT, XGBoost, AdaBoost and BiLSTM in predicting aquatic microbes.

### 3.6. Comparison with the State-of-the-Art Tools

MegaR and MetaML are two recent ML-based metagenomics data analysis tools available in the public domain. A comparison of our proposed model with the said tools revealed a higher accuracy of the proposed method than the abovementioned tools. It is worth mentioning that a comparison was made using a standard test dataset based on which the tools’ accuracies were reported. The reported accuracy of MegaR and MeAML were 66.83% and 66.40%, respectively, whereas the proposed method confirmed an accuracy of 88%.

## 4. Discussion

In metagenomics, the complex environmental genomes of several microorganisms present in a particular niche are analyzed. The metagenome provides a true representation of the microbes in a particular environment. Among the several microbial niches, the rivers are one of the major sinks of beneficial and harmful microbes. A better understanding of the microbial community helps to identify the major pollution sources from harmful microbes and their bioremediation measures to bring the pollution under control [52]. River metagenomics plays a significant role in analyzing microbial population and their diversity. It provides a deep knowledge of microbes in that stretch of river, which can cause skin and other human and animal diseases [53]. The analysis of the river metagenome was previously performed using heuristic approaches, which take much time to analyze the data. Thus, we analyzed the metagenomic datasets collected from two major river ecosystems in India at three locations. Here, we unraveled the microbial diversities in the collected samples and proposed an MLA-based method for annotating unknown samples.

**Figure 7:**
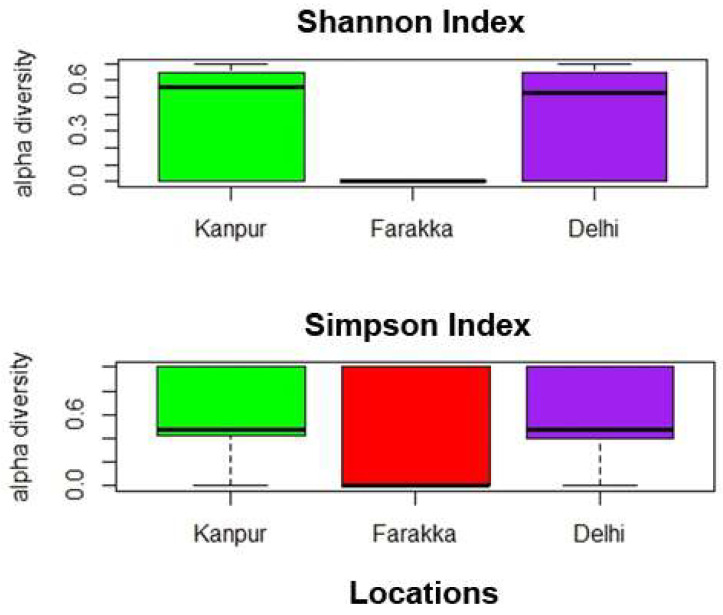
Box plots illustrating α diversity indices (Shannon diversity, Simpson index) for the samples collected from Kanpur, Farakka, and Delhi locations. A lower Shannon index and higher Simpson index represents low diversity.

The river metagenomic dataset was collected from three distinct locations with a comprehensive coverage where the sampling units are located more than a thousand kilometers apart from one another. The assembly statistics confirm suitably long N50 contigs from each location. It is observed from Table 2 that fewer numbers (9771) of scaffolds are generated for the Farakka dataset than the Delhi (69,083) and Kanpur (83,982) datasets, even though the N50 and N90 scores are nearly equal. This could be due to less microbial diversity in Farakka than in the Delhi and Kanpur locations. To validate this assumption, we carried out the αdiversity analysis using *vegan* R-package and represented the diversity in terms of Shanon [54] and Simpson [55] indices (Figure 7), which also shows low microbial diversity at Farraka compared to the other two locations.

Water pollution often affects the microbial diversity of the ecosystem [56]. Pollution can have significant effects on water quality through various mechanisms. According to the US Environmental Protection Agency (https://www.epa.gov/caddis-vol2/ph, accessed on 6 May 2023), certain pollutants can decrease pH levels, which can create an acidic environment that can be harmful to many aquatic organisms. Additionally, pollution can lead to the buildup of dissolved organic matter, which can cause issues such as reduced light penetration and decreased oxygen levels. This decrease in dissolved oxygen can lead to the development of hypoxic or anoxic zones, where the lack of oxygen can have a negative impact on many aquatic species. Furthermore, changes in the chemical composition of water resulting from pollution can affect the diversity and abundance of microbial communities present in the water. These changes can have a cascading effect on the entire aquatic ecosystem, potentially leading to long-term ecological damage. The difference in microbial diversity between Farakka and Delhi/Kanpur could be due to the impact of pollution in these two cities. It is also worth mentioning here that several kinds of leather, thermal and manufacturing industries are present in Kanpur and Delhi, which discharge industrial waste into the rivers, contributing significantly to the water pollution [57,58]. Further, Delhi and its peripheries such as Faridabad and Ghaziabad are also designated as the most polluted cities in the world [59]. The BLAST-based identification of microbes provided a bird’s eye view of microbial diversity in the collected river metagenomic samples. Based on the geographical and environmental conditions, varied microbial diversity was observed at different locations (Table 3). We also observed several microbial genera to be uniquely present at specific locations. Further, the abundance of bacteria at each site was found to be higher as compared to the other classes of microbes. This could also be due to the pollution level in the considered river ecosystems as the bacterial diversity in an aquatic ecosystem is often related to its pollution level [60]. Thus, the microbial diversity in the river ecosystems should be identified and analyzed to take protective measures in improving the health of aquatic animals. The correct identification of microbial diversity also provides information on the water quality, its fitness for agriculture and the pollution status of the river ecosystem. Thus, NGS techniques, along with suitable computational algorithms, are required for developing efficient prediction systems for identifying a correct microbial class from a metagenomic sample. Several clustering and machine learning techniques were applied for metagenomic data analysis [61]. Here, we implemented K-Means clustering for grouping metagenomic scaffolds into five different microbial classes. The classified contigs were used to train various machine learning algorithms to find out the best prediction model that can correctly identify the microbial diversity. We implemented the SVM, RF, GBDT, XGBoost, AdaBoost and BiLSTM for this purpose, and we found that the RF performs consistently well with an acceptable accuracy (88%) irrespective of the location.

The overall accuracies were evaluated by calculating the weighted means of all five classes’ accuracies. However, the tradeoff between the precision, recall, F1 Score and AUC–ROC curve varied from class to class and location to location. Most of the methods were found to perform well with the Delhi location dataset compared to Farakka and Kanpur. This is expected due to the high data quality of Delhi in terms of N50, N90, average scaffold length and the number of scaffolds (Table 2). The RF also performed better, with an accuracy of 88% for the Delhi dataset, which is the highest irrespective of location and method. The violin plot presented in Figure 5 also indicates a consistent accuracy of the RF at different locations with minimal variance in the accuracy. The SVM showed a high variance in the accuracy with the Delhi dataset, whereas the GBDT showed the least deviation. However, the GBDT showed a high variance in the accuracy with the Kanpur dataset. Further, the XGBoost, AdaBoost and BiLSTM showed a high variance in the accuracy with the Farakka dataset, and AdaBoost also showed a high variance with the Kanpur dataset. Further, the RF also confirmed higher performance values of AUC–ROC for all location datasets. Thus, the level of accuracy and consistency of accuracy of the RF in classifying microbial diversity from river metagenomic data suggests that the RF with the considered set of parameters (Table 1) can be a suitable prediction model for correctly classifying the contigs or scaffolds generated from metagenomic samples.

## 5. Conclusions

Metagenomics is a broader field to identify microbial ecology. It plays a significant role in the field of metagenomic research and the industrial community. The experimental identification and characterization of microbes from their niche impact the lives of humans, plants and animals. However, the process is cumbersome, time- and money-consuming. In contrast, computational approaches being complementary to experimental methods allow for the faster characterization and annotation of river microbiome in a particular niche. With the advancement of NGS and machine learning technologies, metagenomic analyses have become easy and convenient. In this study, the role of K-Means clustering for initial binning of the metagenomic scaffolds was explored. Approximately 61% of metagenomic scaffolds from three locations were classified into different microbial clusters. Further, a Random Forest-based classifier with a suitable set of parameterswas proposed for identifying the unknown microbial genus from a metagenomic sample, which is made publicly available at (https://github.com/Nalinikanta7/metagenomics).The proposed model is expected to help the researchers and related stockholders to identify the type of microbes present in a river ecosystem, which, in turn, will help in analyzing the water quality and aquatic health. Accordingly, preventive measures can be taken by the concerned organizations to ensure suitable quality of water for the aquatic health, agriculture and animal health dependent on the river ecosystem.

## Figures and Tables

**Table 1:** Parameters of machine learning algorithms used after a grid search for the best set of parameters.

Classifiers	Parameters		
SVM	C = 10, gamma = 0.001, kernel = ‘rbf’		
RF	max_depth = 25, min_samples_leaf = 2, n_estimators = 25, random_state = 42		
GBDT	n_splits = 10, n_repeats = 3, random_state = 1		
XGBoost	learning_rate = 0.2, n_estimators = 1000, max_depth = 4,min_child_weight = 6, gamma = 0, subsample = 0.8, colsample_bytree = 0.8, objective = ‘binary:logistic’,nthread = 4, scale_pos_weight = 1, seed = 27		
AdaBoost	learning_rate = 0.1, n_estimators=100		
BiLSTM	Layer (type)	Output Shape	Param
	bidirectional (Bidirectional)	(None, 30, 128)	164,864
	bidirectional_1 (Bidirectional)	(None, 128)	98,816
	dense (Dense)	(None, 1)	129
	Total params: 263,809 Trainable params: 263,809 Non-trainable params: 0		

**Table 2:** The metagenomic assembly statistics for three locations obtained using SoapDenovo2.

Parameters	Farakka	Kanpur	Delhi
No. of raw reads	111,809,841	98,019,576	195,606,736
No. of trimmed reads	108,526,822	96,309,665	192,296,837
Number of scaffolds	9771	83,982	69,083
Number of contigs	44,132,563	18,200,983	54,832,125
Total scaffold length(bp)	4,232,650	49,399,583	36,990,293
Average scaffold length	433	588	535
Longest scaffold (bp)	3859	7427	5265
N50	128	110	128
N90	95	91	96

**Table 3:** The microbial diversity showing the abundance of major microbial genus in the Ganga and Yamuna rivers at three different locations.

Microbial Diversity		Ganga		Yamuna
		Farakka	Kanpur	Delhi
Cellular	Bacteria	Streptomyces(400), Pseudomonas(180), Sphingomonas(180), Bradyrhizobium(140), Burkholderia(130), Azospirillum(100)	Pseudomonas(2980), Streptomyces(2910), Burkholderia(1370), Sphingomonas(1320), Bradyrhizobium(1280)	Dechloromonas(3095), Paraburkholderia(2047), Streptomyces(2012), Achromobacter(1359), Acidobacteria(1125), Aeoliella(1063)
	Archaea	Natrinema (258), Nitrososphaera(197), Cenarchaeum(125), Thermococcus(95), Salinigranum(54)	Haloterrigena (395), Aeropyrum (187), Haloarcula(135), Methanothrix(123)	Thermococcus(348), Haloferax(295), Halobacterium(181), Halogeometricum(126)
	Eukaryota	Paracoccus(147), Lobosporangium(159), Parastrongyloides(57), Emiliania(38)	Cyclotella(356), Timema(329), Aspergillus(259)	Gaeumannomyces(124), Phoenix(98), Strongyloides(56)
Non-cellular	Virus	Variovorax(45), Roseovarius(21)	Variovorax(347), Roseovarius(321)	Roseovarius(124), Variovorax(119)

**Table 4:** The overall performance metrics of machine learning algorithms in classifying the microbial diversity in the river metagenomic samples.

Classifiers	Dataset (Location)	Accuracy	Precision	Recall	F1 Score	AUC–ROC
SVM	Farakka	0.76	0.80	0.75	0.80	0.81
	Kanpur	0.78	0.78	0.78	0.78	0.83
	Delhi	0.86	0.81	0.79	0.79	0.86
RF	Farakka	0.85	0.83	0.76	0.80	0.85
	Kanpur	0.84	0.79	0.79	0.79	0.80
	Delhi	0.88	0.85	0.80	0.80	0.87
GBDT	Farakka	0.80	0.80	0.78	0.80	0.79
	Kanpur	0.80	0.80	0.82	0.79	0.80
	Delhi	0.85	0.81	0.79	0.80	0.81
XGBoost	Farakka	0.85	0.79	0.77	0.79	0.75
	Kanpur	0.79	0.76	0.73	0.79	0.81
	Delhi	0.80	0.83	0.79	0.80	0.79
AdaBoost	Farakka	0.77	0.78	0.77	0.80	0.78
	Kanpur	0.79	0.78	0.79	0.77	0.74
	Delhi	0.80	0.84	0.81	0.80	0.77
BiLSTM	Farakka	0.81	0.80	0.75	0.79	0.75
	Kanpur	0.75	0.79	0.80	0.80	0.79
	Delhi	0.79	0.80	0.78	0.78	0.76

**Table 5:** The class-wise performance metrics of machine learning algorithms in classifying the microbial diversity in the river metagenomic sample collected from Farakka.

Classifiers	Multiclass	Accuracy	Precision	Recall	F1 Score	AUC–ROC
SVM	Bacteria	0.75	0.80	0.75	0.79	0.74
	Archaea	0.58	0.78	0.76	0.78	0.72
	Eukaryota	0.65	0.75	0.71	0.69	0.81
	Virus	0.76	0.80	0.75	0.80	0.80
	Others	0.66	0.76	0.74	0.80	0.78
RF	Bacteria	0.70	0.76	0.68	0.80	0.85
	Archaea	0.72	0.72	0.76	0.79	0.85
	Eukaryota	0.69	0.78	0.62	0.78	0.72
	Virus	0.85	0.83	0.77	0.80	0.80
	Others	0.68	0.71	0.77	0.79	0.75
GBDT	Bacteria	0.69	0.80	0.78	0.80	0.59
	Archaea	0.58	0.68	0.82	0.79	0.68
	Eukaryota	0.74	0.78	0.76	0.78	0.72
	Virus	0.80	0.80	0.78	0.80	0.79
	Others	0.77	0.76	0.74	0.80	0.78
XGBoost	Bacteria	0.72	0.79	0.77	0.79	0.75
	Archaea	0.79	0.76	0.73	0.69	0.61
	Eukaryota	0.69	0.68	0.78	0.80	0.75
	Virus	0.82	0.79	0.77	0.79	0.75
	Others	0.74	0.78	0.76	0.78	0.52
AdaBoost	Bacteria	0.77	0.78	0.77	0.80	0.78
	Archaea	0.61	0.78	0.79	0.77	0.64
	Eukaryota	0.69	0.75	0.65	0.77	0.78
	Virus	0.71	0.84	0.81	0.80	0.67
	Others	0.74	0.78	0.76	0.78	0.52
BiLSTM	Bacteria	0.52	0.80	0.75	0.79	0.75
	Archaea	0.75	0.79	0.75	0.71	0.72
	Eukaryota	0.82	0.78	0.77	0.79	0.70
	Virus	0.79	0.80	0.78	0.78	0.71
	Others	0.64	0.79	0.65	0.79	0.74

**Table 6:** The class-wise performance metrics of machine learning algorithms in classifying the microbial diversity in the river metagenomic sample collected from Kanpur.

Classifiers	Multiclass	Accuracy	Precision	Recall	F1 Score	AUC–ROC
SVM	Bacteria	0.79	0.81	0.75	0.80	0.74
	Archaea	0.74	0.78	0.76	0.78	0.72
	Eukaryota	0.78	0.75	0.71	0.69	0.80
	Virus	0.78	0.78	0.78	0.79	0.86
	Others	0.77	0.76	0.74	0.80	0.78
RF	Bacteria	0.78	0.76	0.76	0.78	0.85
	Archaea	0.71	0.79	0.71	0.79	0.80
	Eukaryota	0.74	0.78	0.76	0.78	0.72
	Virus	0.80	0.70	0.69	0.73	0.87
	Others	0.70	0.79	0.73	0.71	0.75
GBDT	Bacteria	0.69	0.80	0.78	0.78	0.79
	Archaea	0.75	0.80	0.82	0.79	0.80
	Eukaryota	0.74	0.78	0.76	0.78	0.72
	Virus	0.81	0.80	0.79	0.70	0.78
	Others	0.77	0.76	0.74	0.72	0.78
XGBoost	Bacteria	0.72	0.75	0.69	0.79	0.75
	Archaea	0.79	0.76	0.73	0.79	0.81
	Eukaryota	0.69	0.68	0.73	0.79	0.79
	Virus	0.78	0.63	0.71	0.70	0.79
	Others	0.74	0.71	0.59	0.72	0.72
AdaBoost	Bacteria	0.77	0.78	0.77	0.71	0.71
	Archaea	0.81	0.78	0.79	0.77	0.74
	Eukaryota	0.69	0.75	0.65	0.77	0.70
	Virus	0.80	0.78	0.78	0.75	0.69
	Others	0.74	0.78	0.76	0.70	0.72
BiLSTM	Bacteria	0.62	0.68	0.75	0.79	0.75
	Archaea	0.75	0.79	0.80	0.80	0.79
	Eukaryota	0.71	0.78	0.77	0.80	0.78
	Virus	0.73	0.77	0.78	0.78	0.76
	Others	0.64	0.72	0.65	0.79	0.74

**Table 7:** The class-wise performance metrics of machine learning algorithms in classifying the microbial diversity in the river metagenomic sample collected from Delhi.

Classifiers	Multiclass	Accuracy	Precision	Recall	F1 Score	AUC–ROC
SVM	Bacteria	0.71	0.80	0.75	0.79	0.74
	Archaea	0.74	0.78	0.76	0.78	0.72
	Eukaryota	0.80	0.75	0.71	0.69	0.80
	Virus	0.82	0.81	0.79	0.79	0.86
	Others	0.77	0.76	0.74	0.80	0.78
RF	Bacteria	0.78	0.76	0.76	0.80	0.85
	Archaea	0.80	0.79	0.79	0.79	0.80
	Eukaryota	0.74	0.78	0.76	0.78	0.72
	Virus	0.84	0.85	0.78	0.80	0.87
	Others	0.82	0.79	0.77	0.79	0.75
GBDT	Bacteria	0.69	0.80	0.78	0.70	0.79
	Archaea	0.55	0.80	0.82	0.79	0.80
	Eukaryota	0.74	0.78	0.76	0.78	0.72
	Virus	0.81	0.81	0.79	0.80	0.81
	Others	0.77	0.76	0.74	0.80	0.78
XGBoost	Bacteria	0.72	0.79	0.77	0.79	0.75
	Archaea	0.79	0.76	0.73	0.79	0.81
	Eukaryota	0.69	0.80	0.78	0.80	0.79
	Virus	0.81	0.83	0.79	0.80	0.79
	Others	0.74	0.78	0.76	0.78	0.72
AdaBoost	Bacteria	0.77	0.78	0.77	0.80	0.78
	Archaea	0.80	0.78	0.79	0.77	0.74
	Eukaryota	0.69	0.75	0.65	0.77	0.79
	Virus	0.80	0.84	0.81	0.80	0.77
	Others	0.74	0.78	0.76	0.78	0.72
BiLSTM	Bacteria	0.78	0.80	0.75	0.72	0.75
	Archaea	0.75	0.79	0.78	0.78	0.72
	Eukaryota	0.77	0.78	0.77	0.78	0.76
	Virus	0.79	0.80	0.78	0.78	0.76
	Others	0.64	0.79	0.65	0.75	0.70

## Data Availability

The metagenomic sequences used in this study were submitted to the NCBI-SRA database under the following accession nos.: SRP190174, SRP190175, SRP189880, SRP191076, SRP191079, SRP191075, SRP191073, SRP191080 and SRP191499for three Kanpur samples (KAN-1, KAN-2, KAN-3), three Farakka samples (FAR-1, FAR-2, FAR-3) and three New Delhi samples (ND-1, ND-2, ND-3), respectively.

## Supplementary Materials

The following supporting information can be downloaded at https://www.mdpi.com/article/10.3390/genes14051082/s1, Table S1: Comparison of different machine learning algorithms used in this study.
