# Supplementary material for: An Improved Machine Learning-Based Approach to Assess the Microbial Diversity in Major North Indian River Ecosystems
Source: Genes (Basel). 2023 May 14;14(5):1082. doi: 10.3390/genes14051082 (PMC10218686; doi:10.3390/genes14051082)
Supplement: Supplementary file 1 [file genes-14-01082-s001.zip › genes-2341533-supplementary.pdf]

Table S1. Comparison of different machine learning algorithms used in this study.

**MLAs**	**Advantages**	**Disadvantages**
Support Vector Machine (SVM)	Works well in high-dimensional spaces, handles both linear and non-linear data, can handle large feature sets	Can be slow for large datasets, sensitive to parameter tuning
Random Forest (RF)	Handles non-linear data, less sensitive to parameter tuning, handles missing data, performs feature selection	Can overfit if too many trees are used, can be slow for large datasets
Gradient Boosting Decision Trees (GBDT)	Handles non-linear data, less sensitive to parameter tuning, handles missing data, performs feature selection	Can overfit if too many trees are used, can be slow for large datasets
XGBoost	Handles non-linear data, less sensitive to parameter tuning, handles missing data, performs feature selection, faster than GBDT	Can overfit if too many trees are used, can be slow for large datasets
AdaBoost	Handles non-linear data, less sensitive to parameter tuning, handles missing data, performs feature selection	Can be sensitive to noisy data
Bidirectional Long Short-Term Memory (BiLSTM)	Handles sequential data well, can learn long-term dependencies, can handle variable-length sequences	Can be slow for large datasets, can be sensitive to noisy data
